# Spontaneous Uterine Rupture and Adenomyosis, a Rare but Possible Correlation: Case Report and Literature Review

**DOI:** 10.3390/diagnostics12071574

**Published:** 2022-06-28

**Authors:** Antonella Vimercati, Miriam Dellino, Cosimina Suma, Gianluca Raffaello Damiani, Antonio Malvasi, Gerardo Cazzato, Eliano Cascardi, Leonardo Resta, Ettore Cicinelli

**Affiliations:** 1 Department of Biomedical Sciences and Human Oncology, Policlinic of Bari, University of Bari, Piazza Aldo Moro, 70100 Bari, Italy; antonella.vimercati@uniba.it (A.V.); damiani14@alice.it (G.R.D.); antoniomalvasi@gmail.com (A.M.); ettore.cicinelli@uniba.it (E.C.); 2 Department of Obstetrics and Gynecology, “Valle d’Itria” Hospital, 74015 Martina Franca, Italy; suma.m@libero.it; 3 Department of Emergency and Organ Transplantation, Pathology Section, University of Bari “Aldo Moro”, Piazza Giulio Cesare 11, 70124 Bari, Italy; g.cazzato3@studenti.uniba.it (G.C.); leonardo.resta@uniba.it (L.R.); 4 Department of Medical Sciences, University of Turin, 10124 Turin, Italy; eliano20@hotmail.it; 5 Pathology Unit, FPO-IRCCS Candiolo Cancer Institute, 10060 Candiolo, Italy

**Keywords:** spontaneous uterine rupture and adenomyosis, morphological uterus sonographic assessment classification

## Abstract

(1) Background: Uterine rupture during pregnancy is a serious obstetric complication with a high incidence of maternal morbidity and mortality. (2) Methods: The present case is a rare event of a uterine rupture occurring in an unscarred uterus in a nonlaboring primigravida woman in the second trimester. The only risk factor in this case was adenomyosis found in the preconceptional phase. (3) Results: The diagnosis of adenomyosis can often be difficult, so patients should be evaluated by a specialized gynecologist. After careful amnestic collection, a gynecological examination and II level ultrasound should be performed in accordance with the Morphological Uterus Sonographic Assessment classification. (4) Conclusions: This evaluation allows us to identify classes of patients at high risk of uterine rupture who, therefore, must be properly informed of the risks both during preconceptional counseling and during pregnancy.

## 1. Introduction

Uterine adenomyosis is defined as the presence of endometrial glands and stroma within the myometrium [1]. One-third of adenomyosis cases are asymptomatic [2]. In the remaining cases, on the basis of structural characteristics and a status of coexisting endometriosis [3], it can be associated with the presence of dysmenorrhea (84%), dyspareunia (26%), metrorrhagia (87%), and chronic pelvic pain (23%) [4]. Furthermore, several studies suggest a possible association of adenomyosis with adverse obstetric outcomes. In women with diffuse or focal adenomyosis, a higher incidence of a small-for-gestational-age (SGA), preterm delivery, preterm pre-labor rupture of membranes, fetal malpresentation, and Cesarean delivery has been reported in the literature [5,6,7,8]. 

On the other hand, the relationship between adenomyosis and uterine rupture (UR) has been only rarely investigated [9]. UR represents a life-threatening obstetrical emergency that is associated with a high maternal and fetal mortality and morbidity rate if not immediately diagnosed and managed. The frequency of UR in pregnant women with a previous Cesarean section is estimated to be between 8.9 and 37.1 per 10,000 births, whereas it is extremely sporadic (between 0.7 and 1.8 per 10,000 births) for patients with an unscarred uterus [10]. We report what we believe to be an exceptionally rare case of spontaneous rupture of an unscarred uterus with adenomyosis in a pregnant woman in the second trimester. The clinical course of the UR in a woman with only adenomyosis and without any additional risk factors for UR is of interest to the medical field. The patient was informed about the use of her personal data for scientific purposes, under the protection of the Privacy Act, and she accepted and signed a related informed consent.

### Case Report

**Figure 1:**
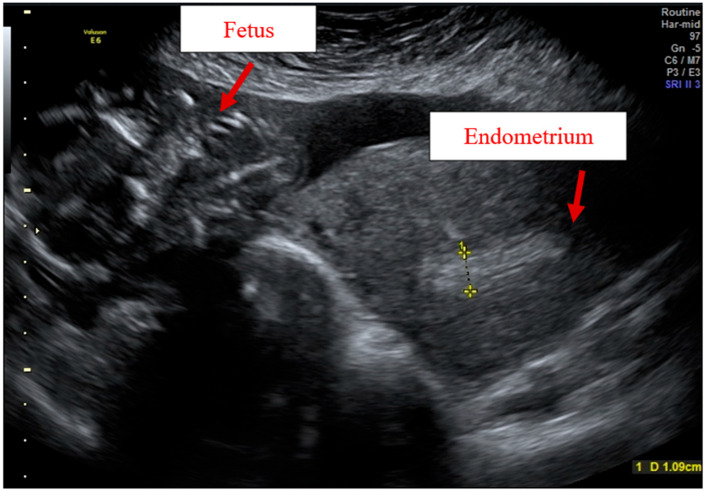
Ultrasound before laparotomy: fetus with amniotic sac completely expelled into the abdomen.

In February 2021, a 27-year-old primigravid woman in the 22nd week of gestation, parity 0/0/0/0 with no relevant medical history, was referred to our clinic for acute abdominal pain. After presenting two times at our obstetric emergency room, the patient was hospitalized. At the admittance ultrasound, the fetal biometrics were normal for the gestational age, the placenta was anterior normo-implanted, and the uterus appeared inhomogeneous due to diffuse adenomyosis. After hospitalization, the patient’s clinical condition worsened. The woman was conscious, but she suddenly became pallid and asthenic, with a BP of 100/50 mmHg, HR of 110 bpm, rhythmic pulse, and an obstetric shock index (OSI) of 0.9 (normal value <1). The laboratory findings upon admission were hematocrit (Hct) 20.8%, hemoglobin (Hb) 6.5 g/dL, white blood cell count (WBC) 17,480 K/mL, and platelet count 203.000 K/mL. The fetal heart rate pattern was evaluated with evidence of severe fetal bradycardia of 70–80 bpm. Due to the maternal deterioration-shock, an ultrasound revaluation was performed, showing the presence of a single fetus and a placenta expelled into the abdomen with free peritoneal fluid, suggesting UR (Figure 1).

**Figure 2:**
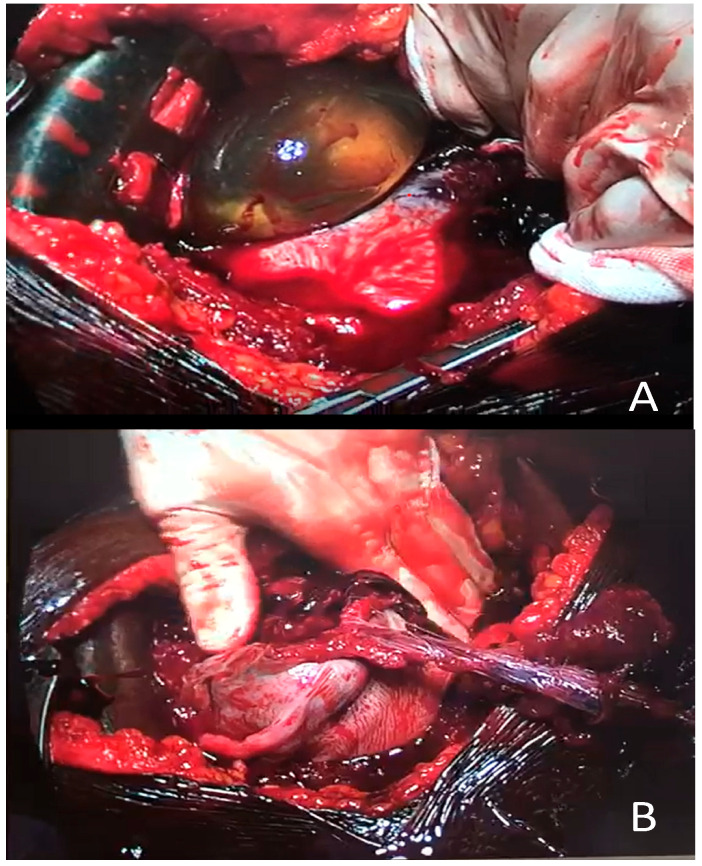
Macro photos after laparotomy: (**A**) Broken uterus with the whole sac containing fetus and placenta expelled in the abdomen; (**B**) extensive and irregular breach on the bottom of the uterus with membranes connected to the expelled sac.

Therefore, the woman was transferred directly to the operating room, and after opening the abdomen, the presence of a massive hemoperitoneum with the whole gestational sac outside of an irregular breach (around 8 cm) on the uterine fundus was detected (Figure 2A,B).

**Figure 3:**
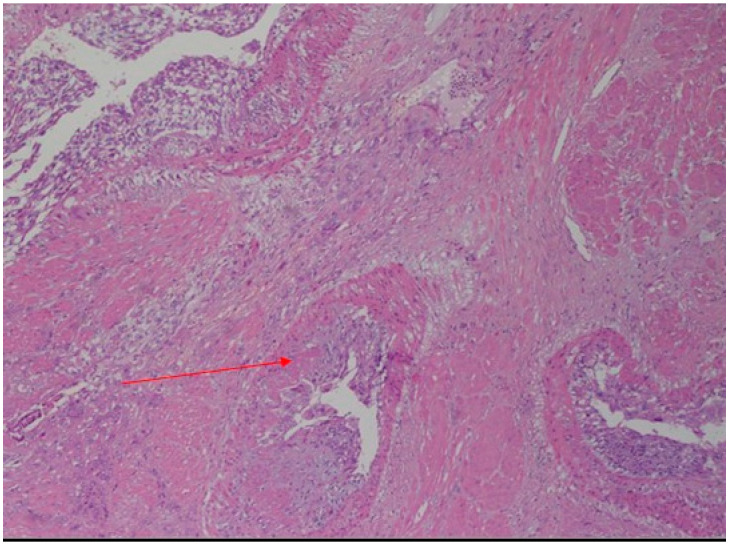
Higher magnification histological micrograph showing the decidualization of the muscle fibers (red arrow). (Hematoxylin-Eosin, Original Magnification: 10×).

After the drainage of 900 mL of blood, the gestational sac containing a stillborn fetus with a weight of 460 g and placenta was detected. The crack was about 8 cm long and located close to the insertion of the right tube. Due to the characteristics of the extensive and irregular uterine laceration, and following the failure to control hemostasis, a hysterectomy with conservation of the adnexa was performed. Moreover, four units of blood and two units of plasma were transfused. The placenta, fetus, and uterus were sent for a pathological examination (the fetus and placenta showed no abnormality for the gestational age). Histological examination of the uterus showed an extensive transmural diffused adenomyosis, especially of the anterior wall of the uterus, with an area of hemorrhage of the surrounding myometrium on the uterine fundus that corresponded with the site of UR (Figure 3).

The rest of the myometrium revealed no evidence of necrosis, fibrosis, or abnormal placentation, but there was diffused adenomyosis. During hospitalization, the hemoglobin level changed from 7.5 g/dL to 6.5 g/dL in the first postoperative day; consequently, two units of packed red blood cells were transfused together with intravenous iron. The patient was also treated with a full course of intravenous antibiotics (cefazolina 3 g /day e.v.). She was discharged on the seventh postoperative day after being informed that the UR was likely to have been caused by adenomyosis, and that no other risk factors had been identified. Moreover, since the patient had suffered from chronic pelvic pain due to severe adenomyosis, it was possible to retrieve an ultrasound evaluation of one year before the current pregnancy from the archives, and all ultrasonographic findings were re-evaluated in accordance with the recent MUSA adenomyosis criteria. Therefore, the “extensive diffuse” adenomyosis pattern was identified showing a diffuse aspect, a cystic lesion, shadow cones, an asymmetrical uterine wall, and a myometrial layer involvement particularly of the anterior-fundic uterine wall middle myometrium (located between the vascular arcade and junctional zone) with a moderate extension. Her gynecologist had not discussed with the patient the potential risks related to extensive adenomyosis and adverse perinatal outcomes.

## 2. Discussion 

Spontaneous UR of an unscarred primigravid uterus is an extremely infrequent obstetric emergency that occurs mainly in the third trimester of pregnancy or intrapartum and rarely in mid-trimester [4,5]. Indeed, Miller et al. have reported that UR in an unscarred uterus in labor is usually mainly connected with the use of uterotonic drugs, multiparity, and malpresentation [10]. On the other hand, Sun et al. have described that sometimes the UR of an unscarred uterus may not be linked with any risk factors. Nikolaou et al. reported that UR could be connected to uterine adenomyosis outside of labor, since it could alter the organization and resistance of the uterine fibers [11]. Nikolaou et al. [12] reported that in nine of the twelve cases of spontaneous UR associated with adenomyosis, the decidualization was histologically highlighted (Table 1). 

Indeed, it was hypothesized that pregnancy hormones could cause two different reactions to the adenomyotic stroma [12]. In the first, superficial foci of adenomyosis, situated at the level of the basal layer of the endometrium, could lead to a little decidualization. In a second pattern, deeper foci of adenomyosis could express a conspicuous decidualization. This interpretation could justify the development of the present case since the large transmural adenomyosis with the marked decidualization and the resultant splaying of the myometrial smooth muscle fibers were probably responsible for the weakening of the myometrium with consequent rupture of the anterior wall and herniation of the amnion across the uterine tear without bleeding. Moreover, in one of the 12 cases, the patient had an endometriosis of the posterior compartment (rectovaginal septum endometriosis), but in this specific case the spontaneous UR began 6 h after delivery. Instead, in another of the 12 cases of spontaneous UR described by Nikolaou, there is no documentation on the presence of pelvic endometriosis but only of a history of infertility.

Subsequently, Indraccolo et al. also described a case of spontaneous UR in a patient with a history of endometriosis (adenomyosis of the uterine posterior wall) and previous laparoscopic surgery due to the presence of adesiolysis for chronic pelvic pain [13]. Recently, Xuqing et al. reported a case of spontaneous UR in a twin pregnancy at 29 weeks and with a history of adenomyosis [14,15,16,17,18]. In this case, uterine overextension due to twinning may have represented a risk factor, considering that the authors described a uterus at 34 weeks of gestation, with no abnormalities in the anterior wall, fundus of the uterus, bilateral tubes, or ovaries (Table 1).To our knowledge, the present case report is the 15th described in the literature in which a correlation between adenomyosis and UR was demonstrated (Table 1); indeed, we have witnessed spontaneous UR in a pregnant woman at 22 weeks and with a clinical and ultrasound diagnosis of preconceptional adenomyosis, particularly of the uterus fundus. The massive UR was located in this area; consequently, a hysterectomy was necessary due to the presence of fibrosis/adenomyosis and due to the extent and irregularity of the lesion that did not allow adequate hemostasis. Therefore, our case highlights the importance of evaluating the diagnosis of spontaneous UR in cases of pregnancies with severe abdominal pain with hypo-volemic shock, uterine tenderness, fetal heart rate bradycardia, and with a history of endometriosis/adenomyosis. After the diagnosis, the management of UR includes emergency laparotomy, with delivery of a viable fetus when possible. It is then important to control the maternal hemorrhage with rapid infusion of crystalloid solutions and a blood transfusion to avoid hemorrhagic shock. If the blood loss is not stopped, a hysterectomy must be immediately performed. The literature has reported that readiness to diagnose and manage rare cases of UR could improve maternal-fetal outcome. Consequently, based on our experience and other authors’ opinions, high-risk patients should be promptly identified and, after counselling, referred to adequate obstetric emergency units for urgent surgical treatment [19,20,21].

### Adenomyosis Ultrasound Diagnosis and Classification

It is important to understand how rare an event spontaneous UR is, but it can be better and earlier diagnosed if an identification of high-risk women is performed. In particular, new risk factors include extended adenomyosis, mainly if it is symptomatic. Since adenomyosis exhibits a diverse and heterogeneous disease spectrum, diagnostic imaging helps to manage patients properly [18]. Indeed, the international Morphological Uterus Sono-graphic Assessment (MUSA) group [21,22,23,24,25] published a consensus on the terminology to apply when describing myometrial lesions. By means of MUSA terminology, the sonographers could describe uterine adenomyosis with seven items that consist of: association of the presence of adenomyosis, discrimination of the location of the adenomyosis, distinction between the focal and diffuse disease, determination between a cystic and non-cystic lesion, examination of myometrial layer involvement, classification of the disease extent as mild, moderate, or severe, and measurement of the size of the lesion. Therefore, for personalized treatments it is important to identify imaging features that can predict the disease severity for each patient. Since the rate of adenomyosis is increasing in women, practicing obstetricians should carefully consider the possibility of obstetrical complications such as UR due to extensive adenomyosis during pregnancy [26,27,28,29,30].

## 3. Conclusions

Adenomyosis is defined as a disorder in which the endometrial glands and stroma are located within the uterine musculature (uterine adenomyomas). The major symptoms of adenomyosis are painful menstruation, chronic pelvic pain, and heavy menstrual bleeding. It is a common disease affecting up to 10% of all women in their reproductive years, and the prevalence increases up to 30–50% in patients who also suffer from infertility and chronic pelvic pain. In addition, as the diagnosis of adenomyosis can often be difficult, in these cases the patient should be evaluated by specialized gynecologists who, after careful amnestic collection, perform a gynecological examination and a II level ultrasound according to the MUSA classification. Therefore, when diagnosing adenomyosis as a risk factor, counseling and care in expert centers should be recommended. Patients should also be informed that prevention is not possible and that, therefore, preterm delivery or at least hospitalization can be considered keeping in mind the rarity of the situation compared with the morbidity and mortality due to preterm delivery. In these patients, in fact, early detection allows us to limit the risk of associated infertility and adverse obstetric outcomes, including UR. These patients should be identified and made aware of the risks of UR related to their condition. As a result, patients with extensive adenomyosis and a high risk of UR may require close follow up and eventual delivery assistance in a center with a neonatal intensive care unit. In addition, during counseling with the patient, she must be informed of the risk that if she feels constant abdominal pain, asthenia, and tachycardia, it may be a UR, and the nearest obstetric emergency room should be prepared. In case of emergency, upon arrival at the hospital the patient herself, being well-informed, will then be able to communicate to the doctors that she has extensive adenomyosis and that she has been informed that this can potentially represent a risk factor for UR. This aids those who see the patient for the first time to not underestimate the case but quickly suspect UR even in a patient with an unscarred uterus and, hence, promptly treat it with a potential improvement of the maternal–fetal outcome.

## Figures and Tables

**Table 1:** Cases of spontaneous uterine rupture in pregnancy due to adenomyosis.

Author	N°	Age	Gravida/Para	Dysmenorrhea	Endometriosis /Adenomyosis	Infertility	Gestational Age	Labor	FHR	Fetus	Apgar	Transfusion	Hysterectomy	Prognosis
Azziz (1986) [5]	1	41	NA/P 10	NA	NA	NA	NA	NA	NA	NA	NA	NA	NA	NA
	1	NA	NA	NA	NA	NA	NA	NA	NA	NA	NA	NA	NA	NA
	1	25	NA/P0	NA	NA	NA	NA	NA	NA	NA	NA	NA	NA	NA
	1	38	NA/P1	NA	NA	NA	NA	NA	NA	NA	NA	NA	NA	NA
	1	33	NA/P0	NA	NA	NA	NA	NA	NA	NA	NA	NA	NA	NA
	1	25	NA/P1	NA	NA	NA	NA	NA	NA	NA	NA	NA	NA	NA
	1	36	NA/P3	NA	NA	NA	NA	NA	NA	NA	NA	NA	NA	NA
Bensaid et al. (1996) [6]	1	22	G1/P1	NA	NA	No	Term	Yes	Severe bradycardia	3000 g	0/2	NA	No	Newborn demise at 3 days
Mueller et al. (1996) [7]	1	30	G1/P0	NA	No	Yes	18	No	NA	NA	NA	NA	Total hysterectomy	Good
Pafumi et al. (2001) [8]	1	30	G3/P2	NA	No	No	37	Yes	NA	2750	8/10	NA	Total hysterectomy	Good
Villa et al. (2008) [9]	1	30	G1/P1	Yes	Rectovaginal septum endometriosis	NA	37 w + 5 d	Yes	Normal	2600	Alive	NA	Rupture 6h postpartum, total hysterectomy	Good
Nikolaou et al. (2013) [12]	1	33	G1/P1	Yes	Ovarian endometriosis	Yes	28	No	Decelerations	1310 g	6/6	6RBC e 3FFP	Subtotal hysterectomy	Good
Indraccolo et al. (2015) [13]	1	37	G2/P0	Yes	Laparoscopy with adhesiolysis for chronic pelvic pain- nodule of the uterine posterior wall and diagnosing an adenomyosis	NA	36	No	Decelerations	NA	9/10	No	No	Good
Li et al. (2021) [14]	1	32	G1/P0- twin pregnancy	Yes	Yes	Yes	29	No	NA	Fetus 1—1440 g Fetus 2—1310 g	Fetus 1:3 /8 5/7	2RBC	No	Good
Present case	1	27	G0/P0	Yes	Yes	No	21 w	No	Severe bradycardia	460 g	Alive	2RBC	Total hysterectomy	Good

Abbreviation: FFP: fresh frozen plasma; FHR: fetal deceleration heart rate; NA: information not available; RBC: red blood cells.

## Data Availability

The datasets generated during the current study are available from the corresponding author on reasonable request.
